# Algerian Tuberculosis Control Program: 60 Years of Successful Experience

**DOI:** 10.7759/cureus.86357

**Published:** 2025-06-19

**Authors:** Kada Bentata, Sofiane Alihalassa, Merzak Gharnaout, Mohamed Abdellatif Bennani, Yahia Berrabah

**Affiliations:** 1 Pulmonology Department, Mostaganem University Hospital, Faculty of Medicine, Abdelhamid Ibn Badis University, Mostaganem, DZA; 2 Pulmonology Department, Blida University Hospital, Faculty of Medicine, Saad Dahlab University of Blida, Blida, DZA; 3 Pulmonology Department, Beni Messous University Hospital, Faculty of Medicine, University of Algiers 1 Benyoucef Benkhedda, Algiers, DZA; 4 Pulmonology Department, Oran University Hospital, Faculty of Medicine, University of Oran 1 Ahmed Ben Bella, Oran, DZA

**Keywords:** active pulmonary tuberculosis, public health, tb-tuberculosis, tuberculin skin test, tuberculosis, tuberculosis control program

## Abstract

Tuberculosis continues to pose a major challenge to the healthcare systems of many countries, particularly those with low to moderate incomes. Since gaining independence in 1962, Algeria has made significant economic and healthcare development strides. Tuberculosis control remains a key focus of its public health strategy. Thanks to the dedicated efforts of its experts and through participation in WHO programs, the prevalence and incidence of tuberculosis, especially the highly contagious pulmonary form, have seen a substantial decrease and have been consistently maintained for several years. This editorial summarizes the progress of tuberculosis control programs in Algeria, detailing their objectives and the positive impact on the epidemiological landscape of tuberculosis over the past decades.

## Editorial

Tuberculosis control represents an important issue in different public health programs established by the WHO and other international and national medical societies. The WHO considers Algeria to be one of the countries that have made notable progress in public health in several areas, including the control of the tuberculosis epidemic. Thanks to the design and implementation of several tuberculosis control programs, the disease has significantly declined in incidence, morbidity, and mortality over the past decades in Algeria [1-3].

After its independence in 1962 and given the socio-economic situation at that time, Algeria faced several challenges, notably that of public health, but also on the economic level, on which the public health situation was very dependent. One of the major public health problems during this period was the tuberculosis pandemic. Few data are available to estimate the epidemiology of tuberculosis in Algeria during the first years of independence. However, data from tuberculin skin test surveys allowed an indirect estimation of the overall disease incidence. After the Second World War, during the initial Bacillus Calmette-Guerin (BCG) vaccination campaigns, studies utilizing tuberculin tests to estimate tuberculosis prevalence reported a reduction in the annual risk of tuberculosis infection, decreasing from 5.01% in 1938 to 4.09% in 1948 [2,4]. Additionally, other surveys estimated an incidence of approximately 300 cases per 100,000 inhabitants [4,5], reflecting the epidemiological situation of tuberculosis in Algeria a few decades before its independence.

After Algeria's independence in 1962, prevalence surveys were conducted across various regions of the country. These surveys primarily relied on tuberculin skin tests among schoolchildren, as advanced screening methods were not yet available. Between 1966 and 1967, studies reported a significant decline in the annual risk of tuberculosis infection, although notable disparities were observed between urban and rural areas. This risk ranged from 2.5% to 4%, depending on the region [2]. This progress coincided with the establishment of the Tuberculosis Office (Bureau de la Tuberculose) in 1964, a unit within the Ministry of Health responsible for managing and monitoring tuberculosis. In 1966, the unit received support from an expert body known as the National Consultative Commission on Phthisiology (Commission Nationale Consultative de Phtisiologie).

This initiative followed recommendations from national experts advocating for the creation of the Algerian League Against Tuberculosis. Affiliated with the three main medical schools of the time, Algiers (the capital), Oran (in the west), and Constantine (in the east), as well as their pneumo-phthisiology departments, this organization was tasked with coordinating tuberculosis control efforts nationwide.

Its mission was based on four primary objectives. First, it aimed to conduct periodic assessments of human and material resources dedicated to tuberculosis control and to develop progressive plans to ensure an equitable distribution of these resources. Second, it sought to evaluate the morbidity and prevalence of tuberculosis in different regions, selected based on the socioeconomic conditions and lifestyle factors. To achieve this, well-trained phthisiology physicians from each region were designated as scientific and medico-social representatives of the central organization. The third key objective was to implement specialized educational programs for medical and paramedical professionals, along with accelerated phthisiology training for the existing nursing staff. Finally, the organization worked to establish a research and documentation center focused on tuberculosis in Algeria and globally. In collaboration with the Ministry of Public Health, it also contributed to formulating a tuberculosis control legislation tailored to the Algerian context. These objectives formed the foundation for Algeria’s various tuberculosis control programs.

The first national anti-tuberculosis chemotherapy program launched in 1966, introduced standardized first-line treatment regimens combining isoniazid, streptomycin, and para-aminosalicylic acid (PAS) for 24 months. A reserve regimen, including ethionamide, cycloserine, kanamycin, or pyrazinamide, was also established. These regimens were provided free of charge and administered on an outpatient basis following brief initial hospitalizations. Priority was given to smear-positive patients [3].

During the same period, therapeutic trials were conducted in Algeria to evaluate a shorter, 12-month tuberculosis treatment regimen. This regimen involved the intermittent twice weekly administration of isoniazid and streptomycin [2].

By the early 1970s, Algeria had a population of approximately 15 million, with a relatively young demographic profile, as 52% of the population was under the age of 20. During this period, the annual incidence of tuberculosis was estimated at 3% [3]. This decade was marked by three pivotal developments in Algeria’s fight against tuberculosis, which significantly shaped the country's tuberculosis control strategy.

One of the major advancements was the sectorization of health services, which facilitated the establishment of anti-tuberculosis units in every health sector across the country. Each unit was equipped with a microscopy laboratory, playing a crucial role in tuberculosis prevention, screening, and treatment. This decentralization improved access to diagnostic and treatment services, ensuring better disease management at the community level.

Another key milestone was the development of the first National Tuberculosis Control Program, introduced in December 1972. This program aimed to integrate anti-tuberculosis activities into all health sectors nationwide, ensuring a unified and systematic approach to tuberculosis control. Additionally, it standardized the evaluation methods for these activities, allowing for more effective monitoring and assessment of tuberculosis prevention and treatment efforts.

Finally, Algeria established a National Tuberculosis Control Laboratory, which became the national reference center for bacteriological and molecular research on tuberculosis [4]. This laboratory played a crucial role in strengthening tuberculosis diagnosis and research, further enhancing the country's ability to combat the disease.

This strategy resulted in positive results in the following years, with annual declarations reported by the WHO showing a decline in the annual incidence of pulmonary tuberculosis in Algeria from 78 cases per 100,000 inhabitants in 1975 to 60 cases per 100,000 inhabitants in 1981 [2]. This progress was reinforced by the establishment of free healthcare, which allowed access for a large part of the population and allowed a more global assessment of the tuberculosis epidemic [3].

During the 1980s, the consolidation of the national tuberculosis control program, the implementation of the short-course treatment regimen, and the regular evaluation of screening and treatment outcomes led to a significant decline in tuberculosis, particularly among children. One of the key components of this program, BCG vaccination, has been a subject of research in Algeria since the 1960s [4]. BCG vaccination has been widely practiced since the establishment of the National Tuberculosis Control Program. A tuberculin skin test survey conducted among schoolchildren between 1980 and 1989 showed a clear decrease in the annual risk of infection, which declined from 0.34% to 0.22%, thanks to the widespread vaccination coverage [4].

During this period, three major advances significantly strengthened tuberculosis control efforts in Algeria. The first was the widespread implementation of BCG vaccination for all newborns, which quickly achieved nearly 90% coverage within a year. This large-scale immunization effort played a crucial role in reducing the risk of tuberculosis infection, particularly among children [3].

Another key achievement was the expansion of microscopy laboratories, which improved tuberculosis diagnosis by enabling bacteriological confirmation in 85% of new pulmonary tuberculosis cases. This advance ensured more accurate detection and treatment of infectious cases, thereby reducing the transmission of the disease [3].

The third major development was the universal adoption of a six-month short-course therapeutic regimen for all forms of tuberculosis across all health sectors. This standardized treatment approach, aligned with global recommendations, significantly improved treatment outcomes and patient recovery rates [3].

The 1990s were among the most challenging years in Algeria's history, marked by severe socioeconomic instability with significant repercussions for public health. In particular, the depletion of rural areas led to an overwhelming influx of people into cities, straining urban health systems that were not adapted to handle such a concentrated population [4]. This situation had a profound impact on the effective management of public health and, consequently, the control of tuberculosis. The period was characterized by frequent shortages of anti-tuberculosis drugs and laboratory supplies, irregular quality control of tuberculosis care, and non-compliance with recommended diagnostic criteria. As a result, there was a considerable increase in the cases of microscopy-negative tuberculosis.

To address this situation, the Ministry of Health tasked the National Committee for the Control of Tuberculosis with revising and updating tuberculosis management guidelines. As a result, the new Tuberculosis Control Manual (Manuel de Lutte Antituberculeuse) was published in 1999 [3]. Among the key objectives set by the program, aligned with WHO recommendations and the DOTS (Directly Observed Treatment, Short-course) strategy, was the detection of at least 70% of contagious pulmonary tuberculosis cases and the successful treatment of at least 85% of these cases [5]. This strategy facilitated the early achievement of the national program’s objectives, which were initially set for 2005.

Between 2000 and 2005, the tuberculosis control strategy focused on two key elements. First, a new information system was implemented to enhance the supervision and evaluation of tuberculosis screening and treatment activities. Second, the organization of BCG vaccination, which had previously been integrated into the Expanded Program on Immunization (EPI), was thoroughly assessed [3].

During this period, and in response to a request from the WHO Africa Region, the National Tuberculosis Committee developed the National Strategic Plan for Revitalizing Tuberculosis Control in Algeria (2001-2005). This coincided with the nationwide adoption of fixed-dose combination (FDC) drugs for the treatment of all forms of tuberculosis, in alignment with WHO guidelines. Another key initiative during this time was the establishment of regional tuberculosis coordination groups to ensure the effective implementation and dissemination of the tuberculosis control relaunch plan and the new recommendations. This period saw stability in the incidence of smear-positive pulmonary tuberculosis to around 26 cases per 100,000 inhabitants [5].

During this period, two new parameters were assessed through national surveys: the prevalence of drug-resistant tuberculosis and the prevalence of HIV among tuberculosis patients. The national survey on tuberculosis resistance revealed a significant decline in primary tuberculosis resistance, decreasing from 8.6% in 1988 to 5.9% in 2002 [6].

Between 2006 and 2015, Algeria’s adoption of the WHO “STOP TB” strategy [7] led the authorities to redefine the sectorization of public health services to enhance the autonomy of primary healthcare services. As a result, hospital structures were reorganized into two types of establishments: Public Hospital Establishments (EPH) and Local Public Health Establishments (EPSP). Additionally, the former Tuberculosis and Respiratory Disease Control Units were restructured into Tuberculosis and Infectious Disease Control Services (SCTMR), serving as the primary referral centers for tuberculosis patients from various healthcare facilities. This new sectorization aimed to strengthen the autonomy of health services while improving the organization of patient care through a clear distribution of responsibilities between SCTMR, specialized hospital services, and university hospitals.

**Figure 1:**
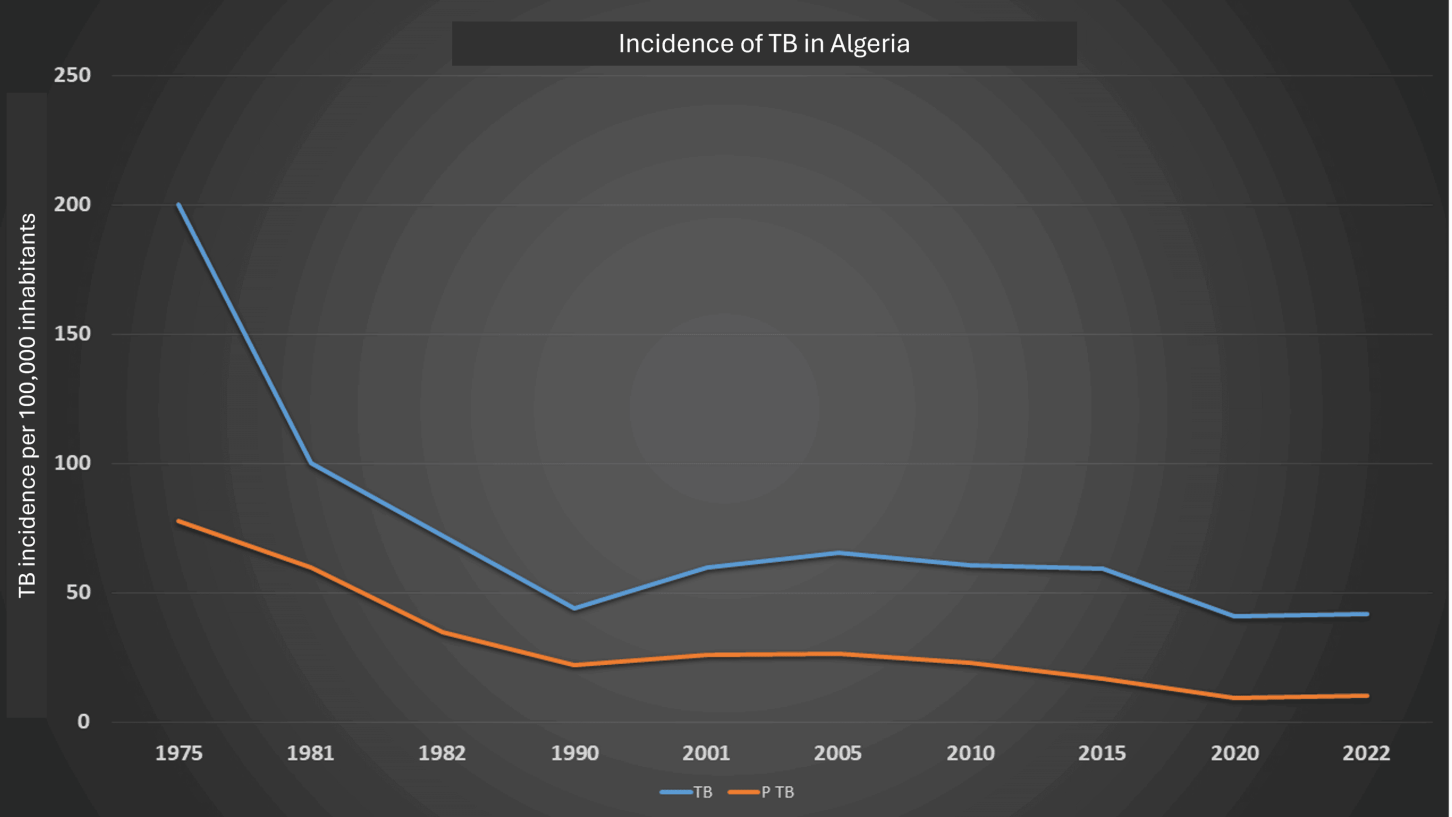
Progression of the incidence of tuberculosis (TB) in Algeria P TB: pulmonary tuberculosis. TB progression in Algeria/100,000 inhabitants. This graphic was created based on data from [3] and freely accessible data from the Algerian National Health Institute website [8].

Since the 2000s, there has been a significant decline in the incidence of contagious pulmonary tuberculosis, with the rate dropping to below 17 cases per 100,000 inhabitants in 2016 (Figure 1).

Experts attribute this decline to the revitalization of the national tuberculosis control program following a period of socioeconomic instability during the 1990s.

The current phase of Algeria’s tuberculosis control strategy aligns with WHO's End TB Strategy, which aims to achieve a 95% reduction in tuberculosis-related deaths and a 90% reduction in its incidence by 2035 compared to 2015 [7]. Achieving these targets will be the next major challenge for the Algerian public health system.
